# Time to relapse in chronic lymphocytic leukemia and DNA-methylation-based biological age

**DOI:** 10.1186/s13148-023-01496-8

**Published:** 2023-05-10

**Authors:** Drew R. Nannini, Rene Cortese, Peter Egwom, Senthilnathan Palaniyandi, Gerhard C. Hildebrandt

**Affiliations:** 1 Department of Internal Medicine, School of Medicine, University of Missouri at Columbia, MA408 Medical Science Building, Columbia, MO 65212 USA; 2 Department of Child Health and Department of Obstetrics, Gynecology, and Women’s Health, School of Medicine, University of Missouri at Columbia, Columbia, MO 65212 USA; 3 Ellis Fischel Cancer Center, University of Missouri at Columbia, Columbia, MO 65212 USA; 4 Division of Hematology and Medical Oncology, School of Medicine, University of Missouri at Columbia, Columbia, MO 65212 USA

**Keywords:** Epigenetic age acceleration, Aging, Chronic lymphocytic leukemia, Chemotherapy, Treatment response

## Abstract

Chronic lymphocytic leukemia (CLL) is a mature B cell neoplasm with a predilection for older individuals. While previous studies have identified epigenetic signatures associated with CLL, whether age-related DNA methylation changes modulate CLL relapse remains elusive. In this study, we examined the association between epigenetic age acceleration and time to CLL relapse in a publicly available dataset. DNA methylation profiling of 35 CLL patients prior to initiating chemoimmunotherapy was performed using the Infinium HumanMethylation450 BeadChip. Four epigenetic age acceleration metrics (intrinsic epigenetic age acceleration [IEAA], extrinsic epigenetic age acceleration [EEAA], PhenoAge acceleration [PhenoAA], and GrimAge acceleration [GrimAA]) were estimated from blood DNA methylation levels. Linear, quantile, and logistic regression and receiver operating characteristic curve analyses were conducted to assess the association between each epigenetic age metric and time to CLL relapse. EEAA (*p* = 0.011) and PhenoAA (*p* = 0.046) were negatively and GrimAA (*p* = 0.040) was positively associated with time to CLL relapse. Simultaneous assessment of EEAA and GrimAA in male patients distinguished patients who relapsed early from patients who relapsed later (*p* = 0.039). No associations were observed with IEAA. These findings suggest epigenetic age acceleration prior to chemoimmunotherapy initiation is associated with time to CLL relapse. Our results provide novel insight into the association between age-related DNA methylation changes and CLL relapse and may serve has biomarkers for treatment relapse, and potentially, treatment selection.

**Supplementary Information:**

The online version contains supplementary material available at 10.1186/s13148-023-01496-8.

## Introduction

Chronic lymphocytic leukemia (CLL) is a mature B cell neoplasm characterized by monoclonal proliferation and accumulation of B lymphocytes in the blood, bone marrow, and lymphoid tissues [1]. Globally, the incidence of CLL has increased over the past several decades and is the most common leukemia in Western countries, with an incidence of 4.2 per 100,000 individuals per year [2, 3]. CLL affects men approximately 2 times more than women, is more common among White individuals, and is most frequently diagnosed in individuals 65–74 years of age with an median age of 70 [4, 5]. As such, given the increasing incidence of CLL, the growing global aging population, and the age-related nature of CLL, examining the underlying determinants and biological processes of CLL may aid in the development of biomarkers for risk stratification, survival, and potentially, response to treatment.

Numerous factors have been associated with CLL, including immunologic, infectious, and anthropometric factors [6]. Molecular and sequencing studies have further identified genomic prognostic factors, including mutations in *TP53* and *IGHV*. [7–9] Moreover, advancements in our understanding of the pathogenesis and prognostic factors have led to improvements in therapies, and subsequent improvement in CLL survival. Fludarabine, cyclophosphamide, and rituximab (FCR) was the first chemoimmunotherapy regimen to induce complete remission and improve overall survival, with bendamustine and rituximab (BR) exhibiting less effectiveness [10]. Recent regimens, including kinase inhibitors targeting Bruton tyrosine kinase (BTK) and phosphatidylinositol 3-kinase, as well as B cell lymphoma 2 (BCL2) antagonists, have transitioned treatments from chemotherapy-based to molecular-targeting due to improved survival and better side effect profiles [10]. Despite the development of efficacious therapies, these targeted agents are expensive, which may be cost-prohibitive, limit access, and reduce adherence to these regimens [11, 12]. As such, identifying additional prognostic markers of CLL treatment response, including CLL relapse, may have implications for prognosis, and potentially, treatment selection.

Aberrations in epigenetic signatures via DNA methylation is a major hallmark in the pathogenesis of cancer [13]. DNA methylation has previously been used to classify CLL patients into prognostic subgroups for overall survival [14–18]. Additionally, age-related DNA methylation signatures, or biological clocks, have been associated with risk of mature B cell neoplasms [19, 20], suggesting these measures of biological age may be useful to predict cancer risk. While these studies demonstrate the risk stratification and prognostic value of epigenetic markers in CLL, the identification of epigenetic features associated with treatment response remains understudied. Although age-related changes to the epigenome in other diseases have previously been associated with disease relapse, providing novel insight of the effect of biological aging on the clinical course of disease [21], these associations have not been well characterized in CLL. As a disease affecting primarily older individuals, these biological clocks may serve as prognostic features for treatment response, including treatment relapse, and potentially, may influence treatment choice. Therefore, we examined the association between four biological clocks as estimated from blood DNA methylation and time to relapse among CLL patients.

## Methods

### Patient samples

We conducted the following analyses using a publicly available dataset with details of the study sample previously described elsewhere [22]. Briefly, 40 CLL patients provided peripheral blood samples at two time points (80 total samples) and guidelines from the International Workshop on Chronic Lymphocytic Leukemia were used to diagnose CLL [23]. The first sample was collected prior to patients’ first chemotherapy treatment (i.e., pretreatment sample) and the second sample was collected after clinically documented relapse (i.e., post-lapse sample). Among the 40 patients, 36 patients received the FCR treatment regimen, 2 received the fludarabine and cyclophosphamide treatment regimen, 1 received the fludarabine, cyclophosphamide, rituximab, and mitoxantrone treatment regimen, and 1 received the BR treatment regimen. Peripheral blood mononuclear cells from peripheral blood were isolated using density gradient separation (tumor load for samples exceeded 51% for CD19+ cells). Negative selection of CD19+ B cells was performed using the RosetteSep B cell enrichment kit (purity for samples > 95% for CD19+ cells)*.* Patients with available documented age at diagnosis were included in downstream analyses (*n* = 38).

### DNA methylation profiling

Patients underwent DNA methylation profiling using the Infinium HumanMethylation450 BeadChip. The R package ENmix was used to perform quality control and data preprocessing using default settings [24]. Probes with a detection *p* < 1 × 10^–6^ and less than 3 beads were defined as low quality. Samples with low quality methylation measurements > 5% or low intensity bisulfite conversion probes (defined as less than 3× standard deviation of the sample bisulfite control intensities below the mean intensity) were removed from further analysis. Samples identified as extreme outliers (defined as less than the 25th percentile minus 3 × interquartile range (IQR) or greater than the 75th percentile plus 3 × IQR; n = 3) and based on the average total intensity value across all probes (defined as unmethylated intensity (U) + methylated intensity (M) or *β*-values [M/(U + M + 100)] were removed. A model-based correction was performed using ENmix and a dye-bias correction was conducted using RELIC [25]. Quantile normalization of U or M intensities for Infinium I or II probes were performed, respectively. Low quality probes and extreme *β*-values across samples were set to missing. After applying these quality control parameters, 35 samples remained for further analysis.

### Epigenetic age calculation

Four epigenetic ages were estimated using the publicly available online DNA methylation age calculator (https://dnamage.genetics.ucla.edu/new). Intrinsic epigenetic age acceleration (IEAA) was estimated using 353 CpG probes and is associated with cell-intrinsic aging [26]. Extrinsic epigenetic age acceleration (EEAA) was estimated using 71 CpG probes and is associated with immunological aging [27]. PhenoAge acceleration (PhenoAA) was estimated using 513 CpG probes and is associated with clinical measures of aging [28]. GrimAge acceleration (GrimAA) was estimated from 1030 CpG probes and is associated with lifespan [29]. Each epigenetic age acceleration (EAA) metric was estimated as the residuals from a linear model of chronological age on epigenetic age.

### Statistical analysis

Multiple linear regression analyses were conducted to examine the association between each EAA metric (independent variable) and time to relapse (dependent variable). Quantile regression was conducted to evaluate the conditional quantiles of each EAA metric on time to relapse. This statistical approach provides a more complete picture of covariate effects on the dependent variable [30]. Relapse status, i.e., early relapse vs late relapse, was generated using the median time to relapse (2.6 years). Logistic regression analyses were performed to examine the association between each EAA metric and relapse status. Receiver operating characteristic (ROC) curves were generated to evaluate the discriminatory ability of each EAA metric by relapse status. To evaluate the discriminatory ability of each EAA metric by sex, stratified analyses by sex were performed. In order to control for confounding of the association between the EAA metrics and time to relapse, chronological age at first treatment, sex, and chemoimmunotherapy regimen were included as covariates in all models. Associations were considered significant if *p* ≤ 0.05. All statistical analyses were performed using SAS Studio (SAS Institute, Inc., Cary, NC, USA).

## Results

### Sample characteristics

Table 1 presents the descriptive characteristics for the 35 patients by relapse status. Overall, the median age of diagnosis among patients was 57 years with a median time to first treatment and time to relapse of 1.5 years and 2.6 years, respectively. Additionally, patients who relapsed early had significantly higher EEAA (*p* = 0.036) and lower GrimAA (*p* = 0.002) compared to those who relapsed late.

**Table 1:** Descriptive statistics of the study sample

	Early relapse	Late relapse	*p*
N, *n* (%)	17 (48.6)	18 (51.4)	–
Female, *n* (%)	6 (35.3)	4 (22.2)	0.458
Age at diagnosis, mean (SD), years	55.4 (10.1)	55.8 (8.9)	0.882
Time to first treatment, mean (SD), years	2.1 (3.3)	2.0 (1.3)	0.875
Time to relapse, mean (SD), years	1.5 (0.5)	5.2 (2.1)	< 0.001
IEAA, mean (SD), years	− 1.6 (17.6)	1.5 (13.8)	0.573
EEAA, mean (SD), years	6.8 (17.8)	− 6.4 (18.0)	0.036
PhenoAA, mean (SD), years	2.3 (16.7)	− 2.2 (22.1)	0.507
GrimAA, mean (SD), years	− 3.2 (6.0)	3.1 (5.2)	0.002

IEAA, intrinsic epigenetic age acceleration; EEAA, extrinsic epigenetic age acceleration; PhenoAA, PhenoAge acceleration; GrimAA, GrimAge acceleration

Table 2 presents the analysis results for the association between epigenetic age acceleration and time to relapse. After adjusting for chronological age at first treatment, sex, and chemoimmunotherapy regimen, EEAA (*p* = 0.011), PhenoAA (*p* = 0.046), and GrimAA (*p* = 0.040) were associated with time to relapse. Specifically, each additional year of EEAA and PhenoAA were associated with a 0.06 [95% CI − 0.11, − 0.02] year and a 0.05 [95% CI − 0.09, 0.00] year decrease in time to relapse, respectively. Moreover, each additional year of GrimAA was associated with a 0.15 [95% CI 0.01, 0.30] year increase in time to relapse. IEAA was not associated (*p* = 0.772) with time to relapse. Similar direction of effects were observed by relapse status during logistic regression (Additional file 1: Table S1). The three most correlated GrimAge surrogate biomarkers of blood plasma proteins with time to relapse were smoking pack-years, plasminogen activation inhibitor 1, and tissue inhibitor metalloproteinase 1 (data not shown).

**Table 2:** Analysis results for the association between epigenetic age acceleration and time to relapse

	*β* [95% CI]	*p*
IEAA	− 0.01 [− 0.07, 0.05]	0.772
EEAA	− 0.06 [− 0.11, − 0.02]	0.011
PhenoAA	− 0.05 [− 0.09, 0.00]	0.046
GrimAA	0.15 [0.01, 0.30]	0.040

Results are adjusted for chronological age at first treatment, sex, and chemoimmunotherapy regimen

Beta coefficients represent the gain in time to relapse for each additional year of epigenetic age acceleration

**Fig. 1:**
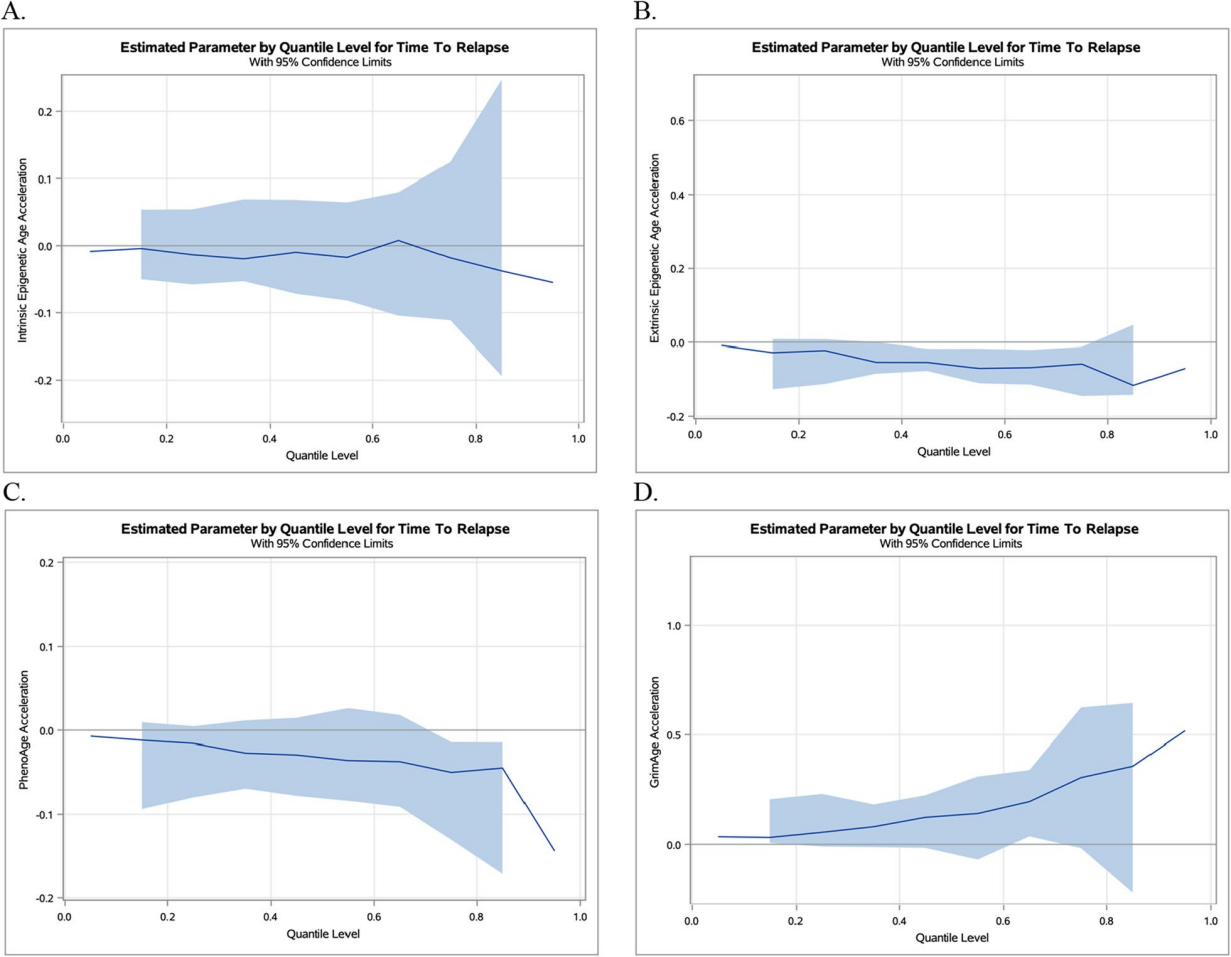
Estimated parameters by quantile with 95% confidence limits for the effect of epigenetic age acceleration on time to relapse. Quantile regression plots for IEAA (**A**), EEAA (**B**), PhenoAA (**C**), and GrimAA (**D**). The x-axis represents the quantile scale, and the y-axis represents the effect of epigenetic age acceleration on time to relapse. Results are adjusted for chronological age at first treatment, sex, and chemoimmunotherapy regimen

Figure 1 presents quantile regression plots for each EAA metric. Regression estimates for 10 quantiles ranging from 0.05 to 0.95 are plotted. As displayed in Fig. 1A, the overall pattern depicts a relatively flat association between IEAA and time to relapse. The effect estimate of IEAA appears to be consistent, with an approximate 0.01-year lower IEAA for nearly all quantiles. In Fig. 1B and C, the effect of EEAA and PhenoAA gradually decreased across the EAA-time to relapse distribution. For example, the effect of EEAA can be four times lower in the upper tail compared to the lower tail of the distribution (i.e., − 0.12-year vs. − 0.03-year, respectively). Similarly, the effect of PhenoAA can be five times lower in the upper tail compared to the lower tail (i.e., − 0.05-year vs. − 0.01-year, respectively). In comparison, the effect of GrimAA gradually increased across the EAA-time to relapse distribution, as displayed in Fig. 1D. The effect of GrimAA can be twelve times greater in the upper tail compared to the lower tail (i.e., 0.36-year vs. 0.03-year, respectively).

**Fig. 2:**
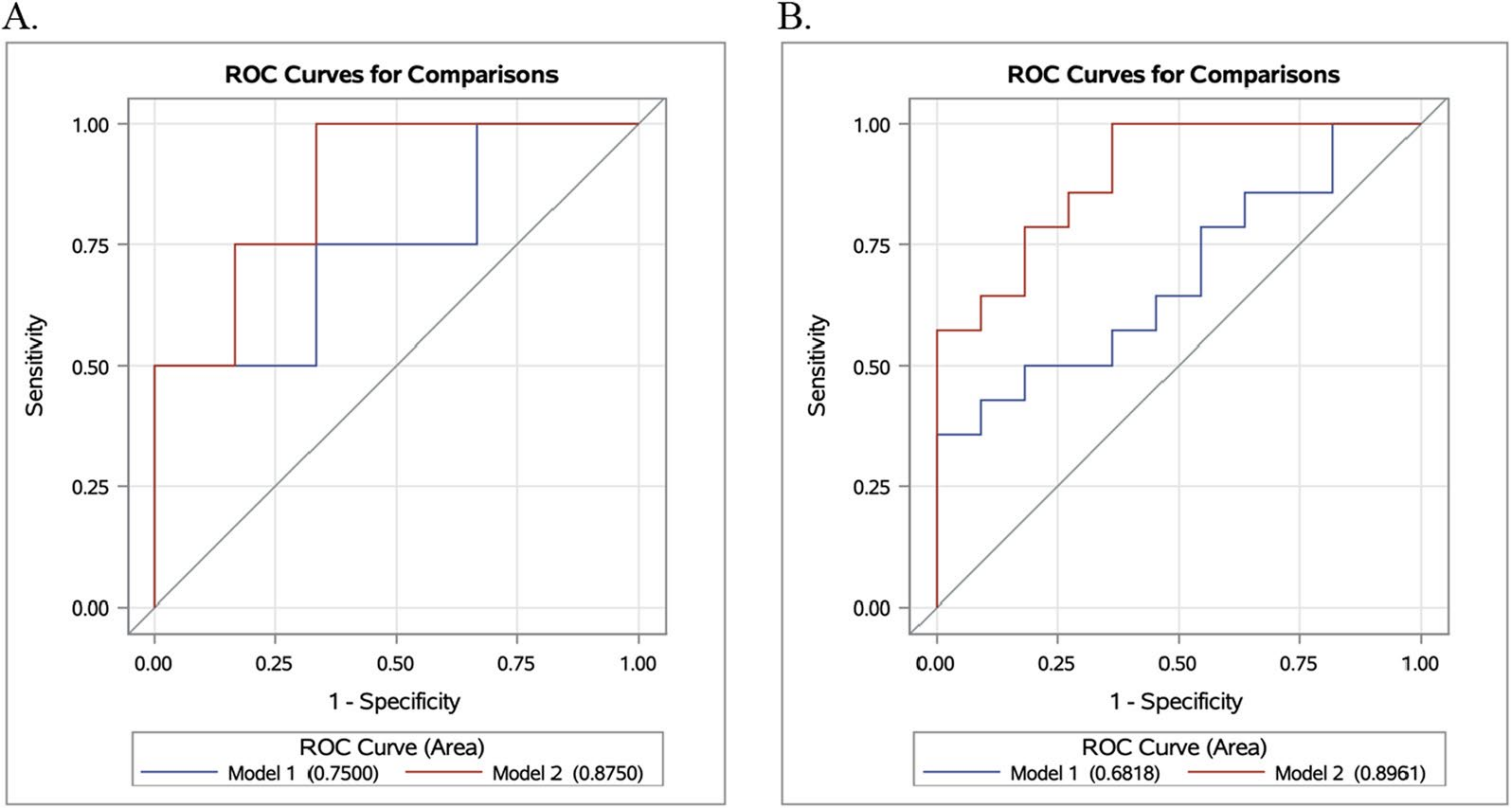
Receiver operating curve comparisons for the discriminatory ability of EEAA and GrimAA between early relapse and late relapse status stratified by sex. Receiver operating curves for female (**A**) and male (**B**) patients. Model 1 evaluates the discriminatory ability of chronological age at first treatment and chemoimmunotherapy regimen. Model 2 evaluates the discriminatory ability of chronological age at first treatment, chemoimmunotherapy regimen, EEAA, and GrimAA. Area under the curve estimates for each model are provided in the figure legend

ROC curves were generated to compare the discriminatory ability of each EAA estimate by relapse status. Compared to chronological age at first treatment, sex, and chemoimmunotherapy regimen (area under the curve [AUC] = 0.650), the addition of each EAA did not statistically improve the discriminatory ability between patients who relapsed early from those who relapsed late (Additional file 1: Table S2; Fig. S1). The addition of EEAA (AUC = 0.788) and GrimAA (AUC = 0.820) moderately increased the discriminatory ability, while IEAA (AUC = 0.631) and PhenoAA (AUC = 0.644) exhibited mild changes in discriminatory ability. The inclusion of EEAA and GrimAA simultaneously further improved classification (AUC = 0.827) between early and late relapse patients (Additional file 1: Table S2; Fig. S2). During sex stratified analyses, IEAA (AUC = 0.917) and EEAA (AUC = 0.917) moderately improved the discriminatory ability between early and late relapse among female patients, while PhenoAA (AUC = 0.750) and GrimAA (AUC = 0.833) mildly improved classification (Additional file 1: Table S3, Fig. S3). In comparison, EEAA (AUC = 0.831) and GrimAA (AUC = 0.883) improved the discriminatory ability between early and late response, while marginal improvement was observed with IEAA (AUC = 0.786) and PhenoAA (AUC = 0.779) among male patients. The inclusion of EEAA and GrimAA simultaneously significantly (*p* = 0.039) improved the discriminatory ability between early and late relapse among male patients and marginally (*p* = 0.361) improved classification among female patients (Additional file 1: Table S3, Fig. 2).

## Discussion

In this study, we observed significant associations between EAA and time to relapse among CLL patients using a publicly available dataset. We observed EEAA and PhenoAA were negatively and GrimAA was positively associated with time to CLL relapse. Furthermore, EEAA and GrimAA improved the discriminatory ability between male patients who relapsed early and patients who relapsed late. These findings suggest age-related changes to the epigenome are associated with response to CLL chemoimmunotherapy and may have implications for selection of therapeutics to minimize relapse, and potentially, improve overall survival.

Epigenetic clocks have been associated with numerous biomarkers, disease risk factors, and health outcomes, with each clock capturing a unique aspect of the aging process. These clocks can be divided into two categories: intrinsic and extrinsic. Intrinsic aging is independent of cell type and partly driven by cellular division, whereas extrinsic aging is influenced by cell type proportion and environmental factors [31]. In our study, we observed associations with extrinsic aging (i.e., EEAA, PhenoAge, and GrimAA) and not intrinsic aging (i.e., IEAA). Specifically, EEAA and PhenoAA were negatively and GrimAA was positively associated with time to CLL relapse. EEAA captures immune system aging weighted by three blood cell types known to vary with chronological age: plasmablasts, native cytotoxic T cells, and exhausted cytotoxic T cells. Moreover, EEAA is positively correlated with estimated abundance of plasmablasts and exhausted cytotoxic T cells, and negatively correlated with native cytotoxic T cells [32]. The increased abundance of CLL cells, derived from naïve B cells, may partially explain the negative association between EEAA and time to relapse, i.e., those with a higher abundance of CLL cells may have greater EEAA and subsequently, a shorter time to relapse. PhenoAA was developed as an estimate for mortality and was generated using nine multisystem clinical chemistry biomarkers, including several hematological features (e.g., lymphocyte percent and white blood cell count) [28]. Similar to EEAA, the association between PhenoAA and time to relapse maybe modulated by these hematological biomarkers, where a higher white blood cell count accelerates aging via PhenoAA and subsequently, a shorter time to relapse. In contrast to EEAA and PhenoAA, GrimAA does not directly include hematological biomarkers, rather eight DNA methylation-based surrogate biomarkers of plasma proteins. Among these surrogate biomarkers, plasminogen activation inhibitor 1 and tissue inhibitor metalloproteinase 1 were moderately, positively associated with time to relapse and these proteins have been proposed as therapeutic targets for myeloid leukemia [33, 34]. The observed positive relationships between these proteins and time to relapse may partially explain the observed positive association between GrimAA and time to relapse, i.e., individuals with higher plasminogen activation inhibitor 1 and tissue inhibitor metalloproteinase 1 activity may be more receptive to treatment and thus have a longer time to relapse, although additional studies are needed to evaluate these associations. In sum, our findings demonstrate several DNA methylation-based biological age estimates are associated with CLL relapse and may serve as potential prognostic biomarkers of treatment response.

To further investigate the association between each EAA metric and time to relapse, we performed quantile regression. Compared to ordinary least squares regression, which models the conditional mean of time to relapse, quantile regression is a statistical approach that enables for a more comprehensive analysis of the conditional distribution of time to relapse for each EAA metric and is beneficial when the change in effect varies by quantile. We observed overall negative associations of EEAA and PhenoAA, and a positive association of GrimAA, on time to relapse during linear regression. During quantile regression, however, the effect of each of these EAA measures varied across the time to relapse-EAA distribution. Specifically, EEAA, PhenoAA, and GrimAA exhibited larger effects at the upper tail of the distributions compared to the lower tail, suggesting patients with more advanced epigenetic aging as captured by these EAA metrics have a larger impact on time to relapse compared to those with less accelerated aging. As such, patients with accelerated extrinsic epigenetic age and PhenoAge and decelerated GrimAge may benefit the most from exploring additional or different therapeutic regimens to obtain a greater time to relapse.

Epigenetics have provided novel insights into CLL classification and prognostic features. While these findings integrate DNA methylation with clinicobiological disease [14–18], the identification of additional biomarkers may aid to further improve predictive abilities for treatment response. In our study, although no single EAA metric was associated with improved discriminatory ability for relapse status, we observed significant improvement among male patients when EEAA and GrimAA were simultaneously included. These results suggest DNA methylation predictors of treatment relapse may differ by patient demographics; a finding not observed in previous studies. Sex differences in response to treatment, treatment side effects, and overall survival have previously been identified, suggesting potential pharmacokinetic differences between females and males [35, 36]. For example, CYP2B6, a hepatic enzyme which metabolizes numerous medications including cyclophosphamide, activity was higher in females compared to males and subsequently, may partially explain differences in treatment outcomes [37, 38]. As such, identifying factors associated with treatment response particularly among patients with earlier treatment relapse and overall survival may have implications for patient management and treatment, such as surveillance and choice of treatment. Furthermore, studies investigating epigenetic factors associated with risk of CLL relapse, compared to time to relapse, may provide additional novel insights and clinical utility of DNA-based biological age metrics. Together, the findings presented here are consistent with previous studies which demonstrated prognostic value of epigenetic signatures for CLL patients and further reveals differences in these features by patient demographics.

This longitudinal study enabled for the examination of pretreatment DNA methylation signatures with CLL relapse. This study, however, is not without limitations. The number of patients included in this study may have resulted in unstable estimates, e.g., confidence intervals and AUC, and may limit the power to detect additional associations between the EAA metrics and time to relapse and as such, additional studies with larger sample sizes are needed to verify the findings presented here. Furthermore, we restricted the analysis to chemoimmunotherapy regimens. These chemoimmunotherapy regimens have been largely replaced by novel targeted therapies, e.g., BTK inhibitors, BCL2 antagonists, and newer generation B cell directed monoclonal antibodies. Therefore, further studies are warranted to investigate whether the observed associations presented here are consistent with more contemporary therapies. Additional patient characteristics were not available and thus, the observed associations may be subject to residual confounding. And lastly, due to the number of EAA metrics and analytic approaches, this study inherently exhibited multiple analyses. Multiple testing correction was not performed due to the analyses being primarily non-independent and to avoid hindering future investigations [39]. Despite these limitations, we observed associations between several EAA metrics and time to CLL relapse, which may have implications for patient stratification and treatment selection.

In conclusion, we observed significant associations between EEAA, PhenoAA, and GrimAA with time to relapse among patients with CLL. We also observed significant improvement in the discriminatory ability by relapse status with the addition of EEAA and GrimAA among male patients. These findings provide novel insights into the association between age-related DNA methylation changes and CLL relapse and highlights the potential role of the aging process in cancer treatment relapse. With the growing aging population and the propensity of CLL to primarily occur in older individuals, identifying biomarkers, including epigenomic markers associated with specific pathologic processes, may further our understanding of therapy resistant cancer and potentially, aid hematological precision medicine efforts to select personalized treatment regimens to improve patient survival.

## Supplementary Information

**Additional file 1**: **Table S1** Analysis results for the association between epigenetic age acceleration and relapse status. **Table S2** Area under the curve estimates and analysis results for the association between epigenetic age acceleration and relapse status. **Table S3** Area under the curve estimates and analysis results for the association between epigenetic age acceleration and relapse status stratified by sex. **Figure S1** Receiver operating curve comparisons for the discriminatory ability of the epigenetic age acceleration estimates between early relapse and late relapse status. **Figure S2** Receiver operating curve comparison for the discriminatory ability of EEAA and GrimAA between early relapse and late relapse status. **Figure S3** Receiver operating curve comparisons for the discriminatory ability of the epigenetic age acceleration estimates between early relapse and late relapse status stratified by sex

## Data Availability

The dataset used in this analysis is available in the EBI repository, https://www.ebi.ac.uk/arrayexpress/experiments/E-MTAB-7575, reference number E-MTAB-7575.
