# Supplementary material for: Time to relapse in chronic lymphocytic leukemia and DNA-methylation-based biological age
Source: Clin Epigenetics. 2023 May 10;15:81. doi: 10.1186/s13148-023-01496-8 (PMC10170738; doi:10.1186/s13148-023-01496-8)
Supplement: Supplementary file 1 — Additional file 1: Table S1 Analysis results for the association between epigenetic age acceleration and relapse status. Table S2 Area under the curve estimates and analysis results for the association between epigenetic age acceleration and relapse status. Table S3 Area under the curve estimates and analysis results for the association between epigenetic age acceleration and relapse status stratified by sex. Figure S1 Receiver operating curve comparisons for the discriminatory ability of the epigenetic age acceleration estimates between early relapse and late relapse status. Figure S2 Receiver operating curve comparison for the discriminatory ability of EEAA and GrimAA between early relapse and late relapse status. Figure S3 Receiver operating curve comparisons for the discriminatory ability of the epigenetic age acceleration estimates between early relapse and late relapse status stratified by sex [file 13148_2023_1496_MOESM1_ESM.docx]

**Supplemental Table 1.** Analysis results for the association between epigenetic age acceleration and relapse status

|  | OR [95% CI] | *p* |
| --- | --- | --- |
| IEAA | 1.00 [0.96, 1.05] | 0.819 |
| EEAA | 0.96 [0.92, 1.00] | 0.059 |
| PhenoAA | 0.99 [0.95, 1.03] | 0.525 |
| GrimAA | 1.21 [1.02, 1.42] | 0.025 |

Results are adjusted for chronological age at first treatment, sex, and chemoimmunotherapy regimen.

Odds ratios represent the odds of relapse for each additional year of epigenetic age acceleration.

**Supplemental Table 2.** Area under the curve estimates and analysis results for the association between epigenetic age acceleration and relapse status

|  | AUC | | *p* |
| --- | --- | --- | --- |
|  | Model 1 | Model 2 |  |
| IEAA | 0.650 | 0.631 | 0.632 |
| EEAA | 0.650 | 0.788 | 0.166 |
| PhenoAA | 0.650 | 0.644 | 0.916 |
| GrimAA | 0.650 | 0.820 | 0.097 |
| EEAA + GrimAA | 0.650 | 0.827 | 0.075 |

Model 1 evaluates the discriminatory ability of chronological age at first treatment, sex, and chemoimmunotherapy regimen. Model 2 evaluates the discriminatory ability of chronological age at first treatment, sex, chemoimmunotherapy regimen, and epigenetic age acceleration.

**Supplemental Table 3.** Area under the curve estimates and analysis results for the association between epigenetic age acceleration and relapse status stratified by sex

|  | AUC | | *p* |
| --- | --- | --- | --- |
|  | Model 1 | Model 2 |  |
| Females |  |  |  |
| IEAA | 0.750 | 0.917 | 0.243 |
| EEAA | 0.750 | 0.917 | 0.129 |
| PhenoAA | 0.750 | 0.750 | 1.000 |
| GrimAA | 0.750 | 0.833 | 0.243 |
| EEAA + GrimAA | 0.750 | 0.875 | 0.361 |
|  |  |  |  |
| Males |  |  |  |
| IEAA | 0.682 | 0.786 | 0.320 |
| EEAA | 0.682 | 0.831 | 0.155 |
| PhenoAA | 0.682 | 0.779 | 0.272 |
| GrimAA | 0.682 | 0.883 | 0.071 |
| EEAA + GrimAA | 0.682 | 0.896 | 0.039 |

Model 1 evaluates the discriminatory ability of chronological age at first treatment and chemoimmunotherapy regimen. Model 2 evaluates the discriminatory ability of chronological age at first treatment, chemoimmunotherapy regimen, and epigenetic age acceleration.

**Supplemental Figure 1.** Receiver operating curve comparisons for the discriminatory ability of the epigenetic age acceleration estimates between early relapse and late relapse status

1. B.


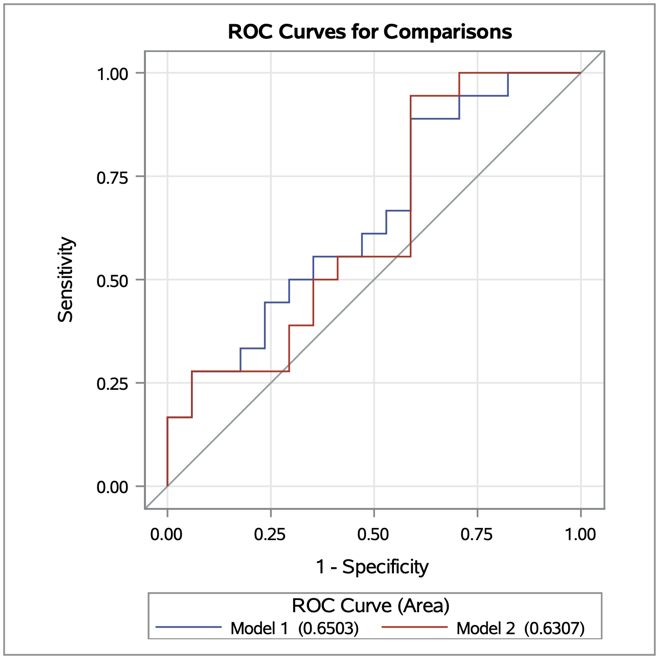

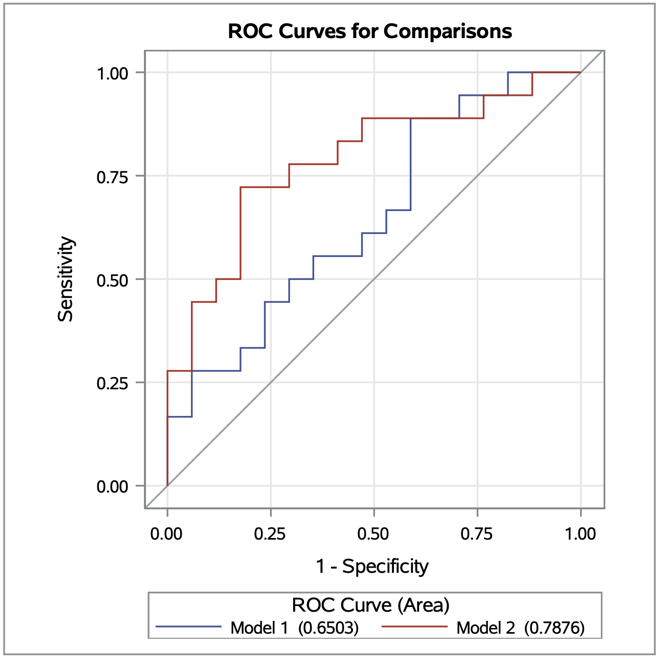


C. D.


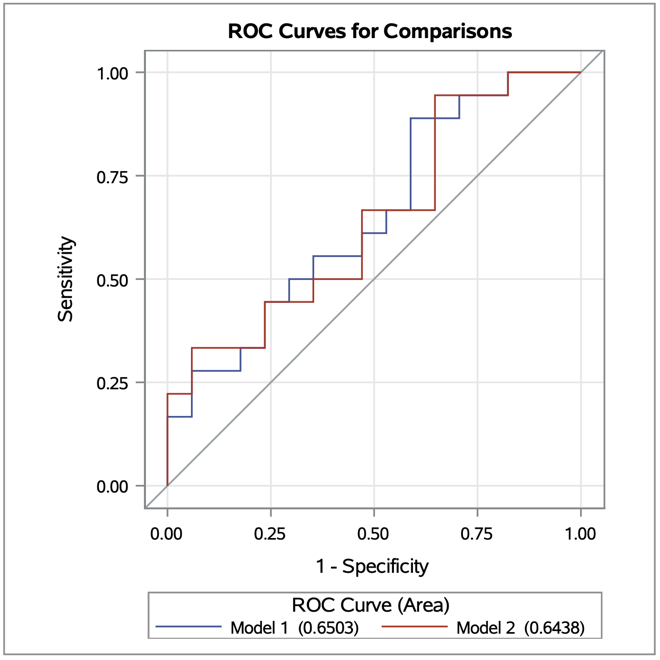

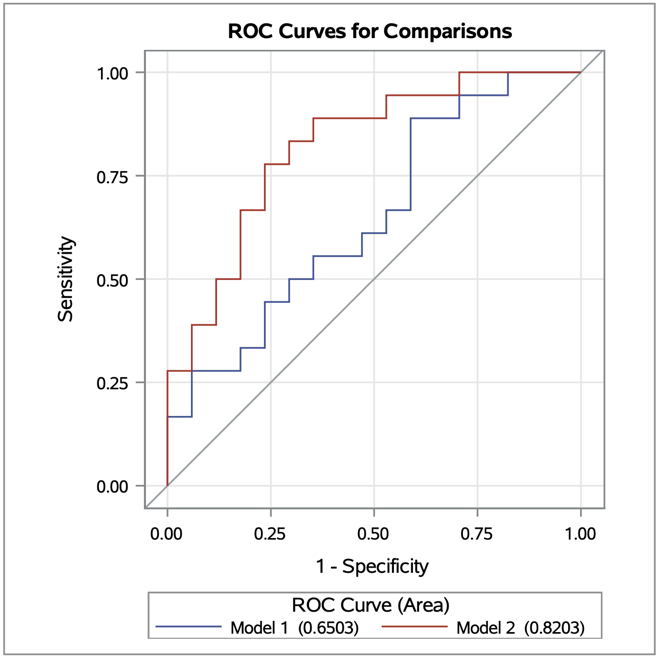


Receiver operating curves for IEAA (A), EEAA (B), PhenoAA (C), and GrimAA (D). Model 1 evaluates the discriminatory ability of chronological age at first treatment, sex, and chemoimmunotherapy regimen. Model 2 evaluates the discriminatory ability of chronological age at first treatment, sex, chemoimmunotherapy regimen, and epigenetic age acceleration. Area under the curve estimates for each model are provided in the figure legends.

**Supplemental Figure 2.** Receiver operating curve comparison for the discriminatory ability of EEAA and GrimAA between early relapse and late relapse status


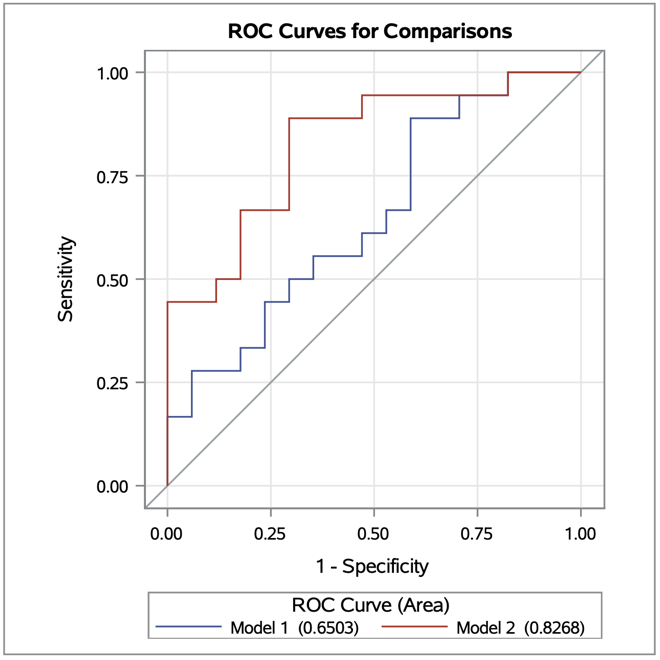


Model 1 evaluates the discriminatory ability of chronological age at first treatment, sex, and chemoimmunotherapy regimen. Model 2 evaluates the discriminatory ability of chronological age at first treatment, sex, chemoimmunotherapy regimen, EEAA, and GrimAA. Area under the curve estimates for each model are provided in the figure legend.

**Supplemental Figure 3.** Receiver operating curve comparisons for the discriminatory ability of the epigenetic age acceleration estimates between early relapse and late relapse status stratified by sex

1. B.


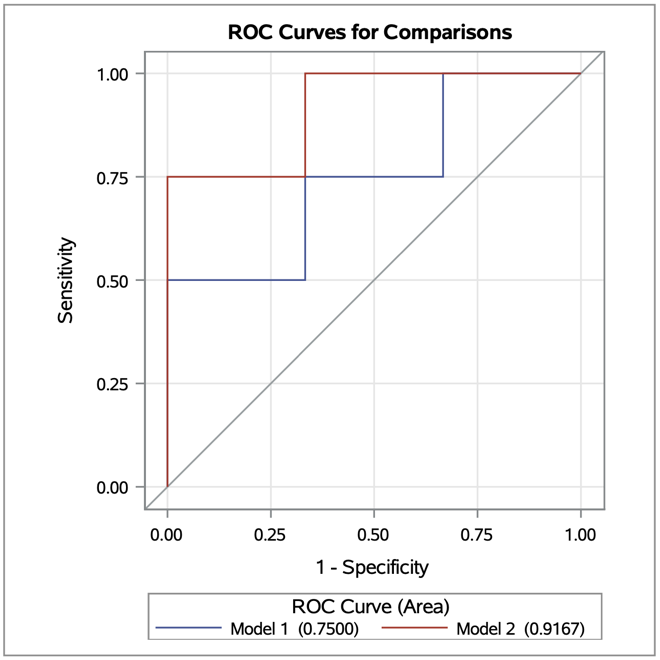

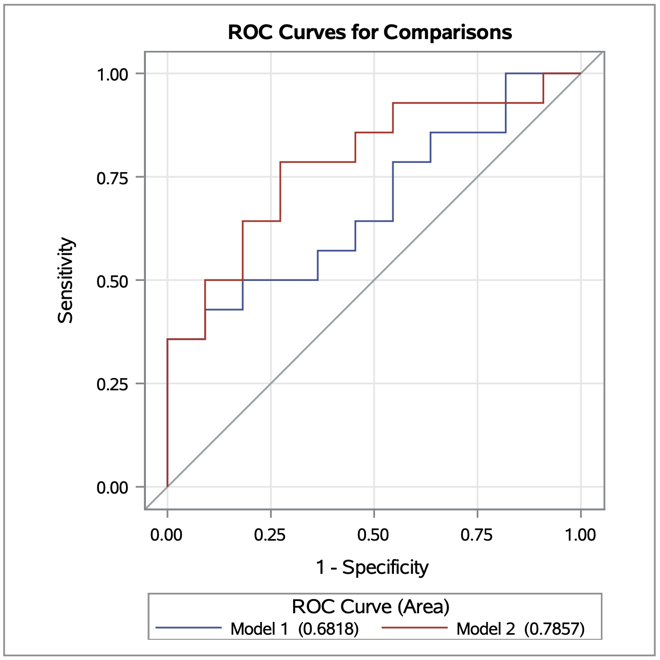


C. D.


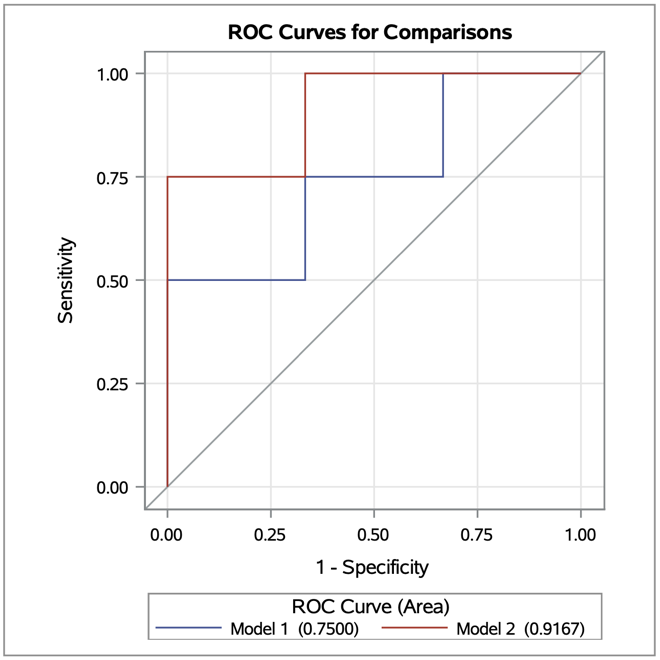

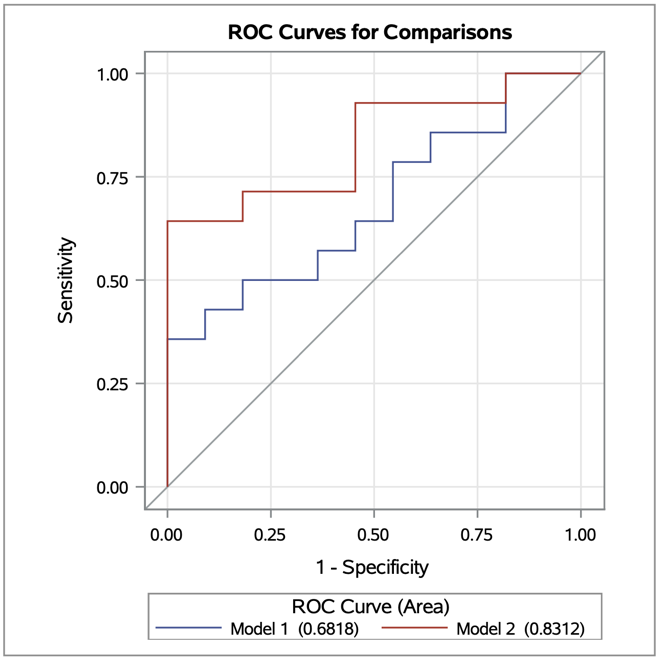


E. F.


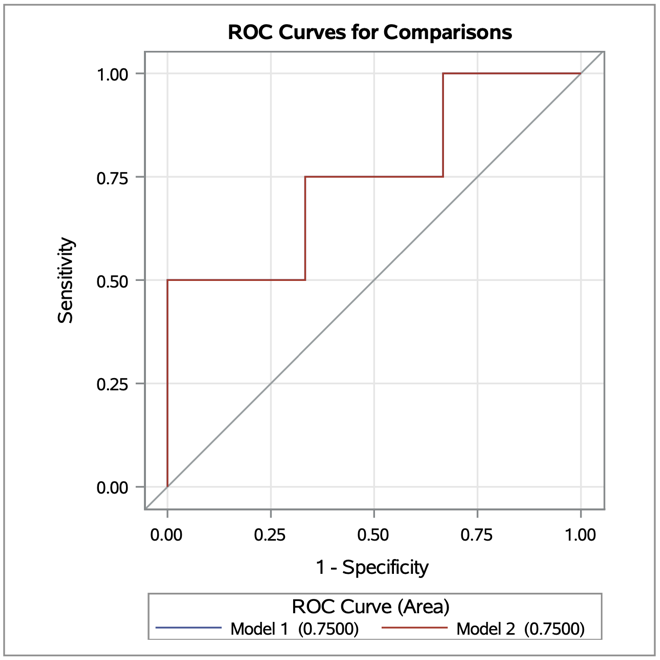

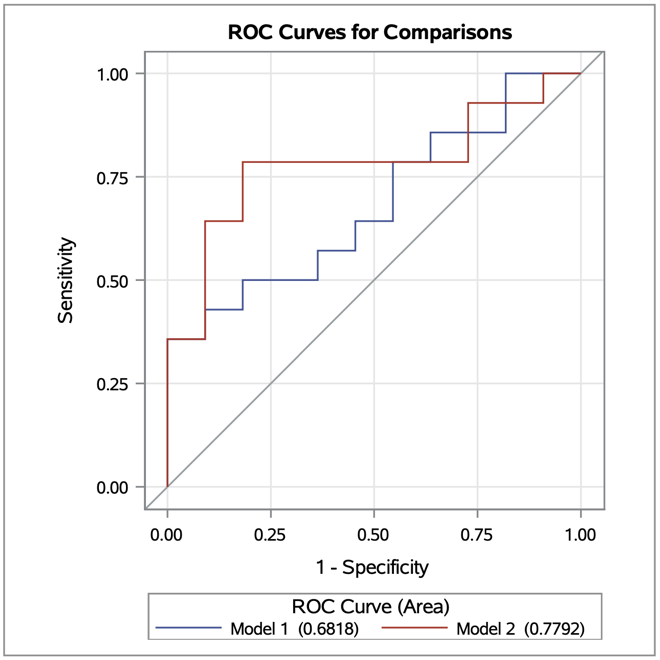


G. H.


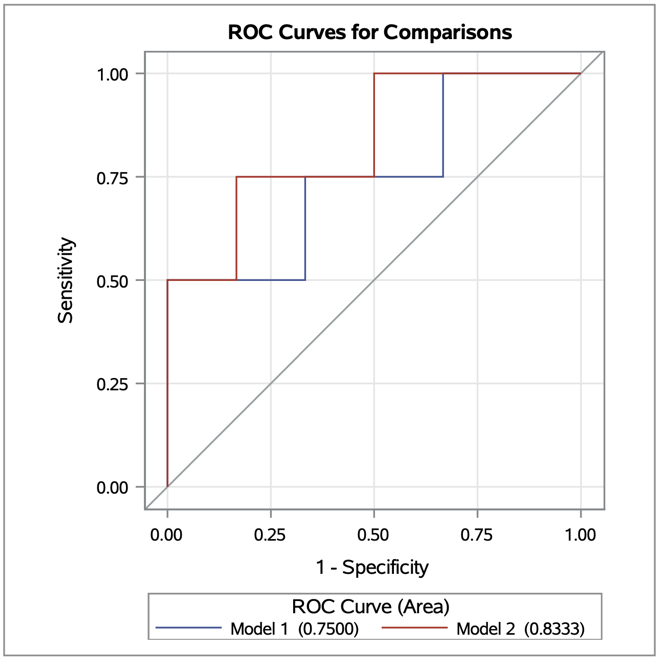

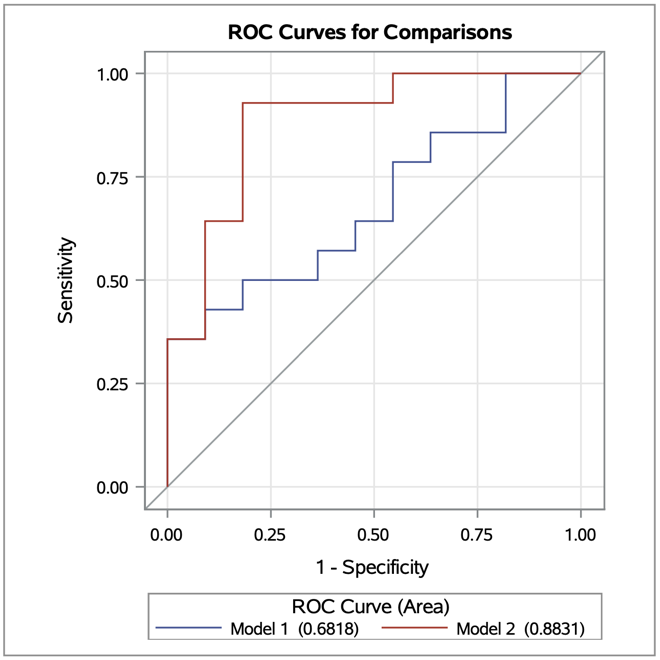


Receiver operating curves for IEAA (A-B), EEAA (C-D), PhenoAA (E-F), and GrimAA (G-H) for female and male patients, respectively. Model 1 evaluates the discriminatory ability chronological age at first treatment and chemoimmunotherapy regimen. Model 2 evaluates the discriminatory ability of chronological age at first treatment, chemoimmunotherapy regimen, and epigenetic age acceleration. Area under the curve estimates for each model are provided in the figure legends.
